# Femoral Malunion and Its Correction: A Review

**DOI:** 10.3390/medicina61112050

**Published:** 2025-11-17

**Authors:** Rahul Vaidya, Matthew Mazur, Ihunanya Agomuoh, David Abdelnour, Magd Boutany, Robert Teitge

**Affiliations:** 1 Department of Orthopedic Surgery, Detroit Medical Center, Detroit, MI 48201, USA; rahvaidya2012@gmail.com (R.V.); mmazu@med.wayne.edu (M.M.); rteitge@med.wayne.edu (R.T.); 2 School of Medicine, Wayne State University, Detroit, MI 48201, USA; abdelnourdavid@wayne.edu (D.A.); mboutany@umich.edu (M.B.)

**Keywords:** femoral malunion, corrective osteotomy, limb deformity, intertrochanteric osteotomy, clamshell osteotomy, limb length discrepancy, rotational deformity, orthopedic trauma, femur reconstruction

## Abstract

*Background and Objectives*: Femoral malunion, defined as healing of a femoral fracture in an anatomically incorrect position, can lead to significant biomechanical and functional impairment despite modern fixation techniques achieving union rates near 99%. The lack of a universal definition and standardized management approach continues to hinder optimal outcomes. This review aims to synthesize the literature on the causes, clinical presentation, radiologic assessment, surgical indications, corrective procedures, and outcomes of femoral malunion to guide clinical decision-making and future research. *Materials and Methods*: A narrative review of peer-reviewed orthopedic literature was conducted, focusing on adult femoral malunions across anatomical regions. Articles detailing deformity thresholds, imaging modalities, corrective osteotomies, and fixation strategies were included. Particular emphasis was placed on region-specific deformities—femoral head, neck, intertrochanteric, diaphyseal, and distal femur—and their corresponding surgical correction methods, including valgus intertrochanteric osteotomy, clamshell osteotomy, and lengthening with external or magnetic intramedullary devices. *Results*: Malunion most commonly presents as angular, rotational, or length deformity, with thresholds of >5–10° angulation, >10° rotation, or >1–2 cm shortening being clinically significant. Patients may experience pain, limp, gait asymmetry, and early-onset arthritis. Corrective techniques tailored to the anatomical site yield favorable results: valgus intertrochanteric osteotomy restores leg length and alignment; diaphyseal malunions respond well to single- or multi-plane osteotomies with internal fixation or gradual correction; distal femoral malunions often require multiplanar osteotomy to reestablish the joint line. Most series report high union rates and functional improvement, though complications such as infection and hardware failure may occur. *Conclusions*: Femoral malunion remains a complex but treatable condition. Successful outcomes rely on accurate deformity characterization, patient-specific surgical planning, and restoration of mechanical alignment. Standardized deformity criteria and long-term functional outcome studies are needed to refine management algorithms and improve patient care.

## 1. Introduction

Surgical fixation of femur fractures in adults typically yields favorable clinical outcomes, with modern intramedullary nailing techniques resulting in healing rates as high as 99% [1,2,3,4,5]. The rate of malunion, however, has been estimated to be roughly 30% (often due to malrotation) while the true incidence is unknown [6,7,8,9,10,11,12,13,14,15,16]. This is due to a lack of a clear-cut definition of symptomatic malunion of femur fractures and/or poor postoperative evaluation for deformity parameters [14]. Radiographic malunion of the femur may be asymptomatic or manifest as gait disturbance, pain, or result in devastating long-term consequences such as joint or back pain, as well as early-onset arthritis [6,9,10,11,12,13].

The purpose of this narrative review is to characterize symptomatic femoral malunion in the adult patient. This includes the causes, radiological parameters, indications for surgery, surgical options, and clinical outcomes associated with treating femoral malunions. By doing so, we aim to develop a more thorough understanding of this problem and inspire future work towards establishing universally accepted clinical treatment guidelines and goals for acceptable treatment.

## 2. Materials and Methods

**Figure 1:**
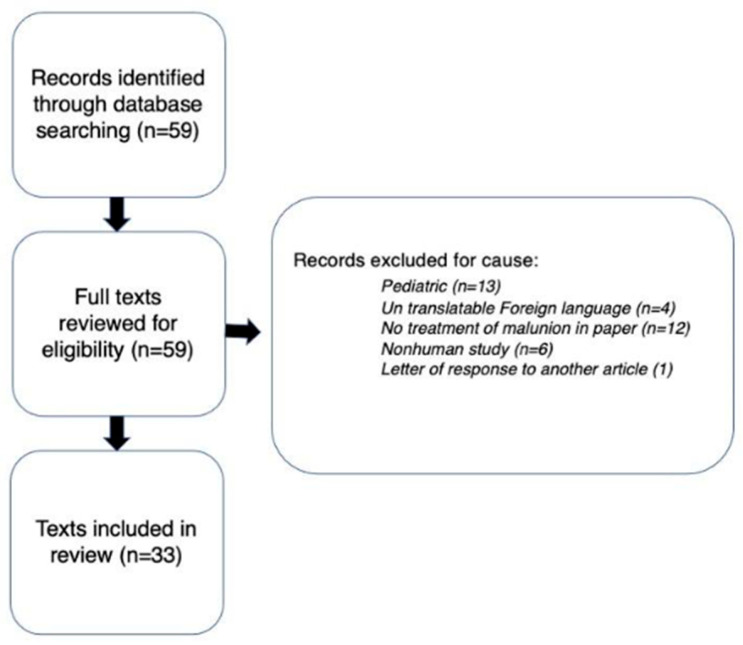
Flowchart displaying methodology for literature review.

An extensive search was conducted on PubMed with the key terms “femur” OR “femoral”, AND “malunion”, AND “osteotomy” OR correction to be included in the article title. At the time, the literature search yielded a total of 59 relevant articles available for review. Four of the articles were excluded based on being written in a non-English language with no available translation. Thirteen articles were discarded because they only included pediatric patients. Six articles were excluded for utilizing non-human subjects. One was excluded as it was a response letter to another article. This left a total of 33 articles. Referential analysis provided additional relevant research material (Figure 1). Every article included was in the format of either a case series (Level 4 evidence), or a case report (Level 5 evidence).

## 3. Definition of Femur Fracture Malunion

Femoral malunion may be described both radiographically and clinically. While the radiographic definition may be more precise, clinical definitions may be more applicable to practice. In his case study (1931), Dr. Patterson provided the broad definition of femoral malunion as any incidence where the bony union of fractured femoral fragments does occur, but in a manner that seriously interferes with function [17]. His description included cases with clinically significant femoral shortening (leg length discrepancy, LLD > 1 ½ inches or 3.81 cm), angulation (degree not defined), malrotation (in only one instance but admittedly due to lack of good records), and joint stiffness. These measurements were taken from direct visualization of fracture fragments [17]. As our treatment of femoral fractures has evolved and improved, so has our definition of acceptable deformity. For example: despite a historical description of 3.81 cm (1.5 inches) as an acceptable LLD, most papers now site shortening of only 1–2 cm of postoperative LLD as acceptable. The current literature defines femoral malunion as an angular deformity >5–10 degrees, a rotational malalignment of >10 degrees, or shortening of >1–2 cm that is symptomatic. These criteria are not widely applicable to each anatomic location within the femur [4,18,19,20,21,22,23,24,25,26]

### 3.1. Cause of Femoral Malunion

There are several causes of femur fracture malunion, many of which are preventable. The most frequent causes include errors in initial fracture treatment due to prolonged timing to diagnosis, improper fracture reduction, inappropriate fixation method, and failure to maintain proper fracture fragment positioning during healing. These may all be attributed to surgeon inexperience or errors [7,8]. Other factors include patient-specific qualities such as noncompliance, comorbidities (diabetes, compromised vascular supply, poor immune response) and infection at or around the fracture site [9,10,11,12,13,17,19,20]. These factors more often lead to nonunion, but can also lead to bony healing with failure to maintain or achieve reduction in the deformity, resulting in a malunion. Less common causes include non-operative treatment and failure of a surgical implant [18,21].

### 3.2. Evaluation

Clinical evaluation includes a thorough history to identify potential causes of the malunion, and risk factors that may impair bone healing or affect treatment. Physical examination should include a recording of both real (measured, objective) and apparent (patient-reported, subjective) LLD, Galeazzi test to determine femoral and tibial contributions to LLD, comparison of hip rotation (either prone or supine, with hip at 90–90 position), evaluation of knee ligamentous integrity, identification of any tibial deformity or joint contractures, description of surrounding soft tissue envelope, and a full neurological exam for deficits which could be aggravated by surgical correction. As opposed to the relatively superficial tibia bone, the femur is robustly covered by soft tissue and can only be visualized at the knee. A physical exam often affords an accurate analysis of tibial deformity and its contribution to LLD. This, in turn, allows areas for correction to be identified much more easily. Conversely, the femur’s length and version are easily masked by hip or pelvic deformities, hip subluxation or dislocation, hip stiffness, and occasionally even tibial deformities. This necessitates a thorough radiographic assessment for all patients. Gait should be assessed, keeping in mind that video-recordings of the patient’s gait can be an extremely valuable tool in characterizing dysfunction as well as treatment outcome.

**Figure 2:**
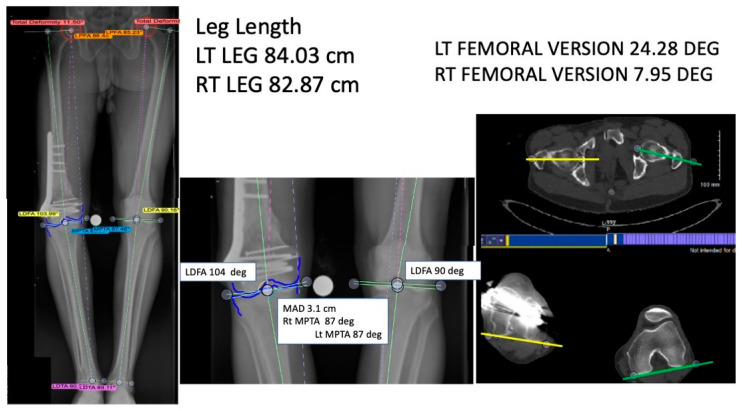
Radiographic and computed tomography assessment of femoral malunion. The left image displays a full-length standing radiographs and CT scans demonstrating deformity analysis in a patient with femoral malunion. In the middle image, mechanical axis deviation (MAD), lateral distal femoral angle (LDFA), and medial proximal tibial angle (MPTA) are measured to evaluate coronal alignment and leg length discrepancy (left leg: 84.03 cm; right leg: 82.87 cm). The Axial CT images on the right show femoral version differences (left 24.28°, right 7.95°), highlighting rotational malalignment. These imaging modalities assist in quantifying angular, rotational, and length deformities prior to corrective planning.

Prior to providing any form of treatment for femoral malunion, the deformity must be clearly defined. For example: Is there simply an angular deformity in the coronal or sagittal plane, or is it instead seen in an off-axis AP/lateral view? Is there malrotation, shortening, or translation? In earlier years, standard physical examination and radiographs were considered sufficient to guide a reasonable treatment plan. Advancements in diagnostic modalities through the years have allowed the evaluation and treatment of femoral malunion to evolve. These modalities include the use of full length standing X-rays (either single or double leg stance) with size markers for coronal alignment parameters and mechanical axis deviation (MAD), use of computed tomography (CT) scans for rotational malalignment and 3D deformity evaluation [22] (Figure 2), and use of magnetic resonance imaging (MRI) scans for joint contractures, ligamentous tears, and nerve or vascular entrapment. In many instances there is a normal contralateral limb for comparison, and when there is not, several authors have established normal coronal, sagittal, and axial alignment parameters [16,22] (Supplemental Materials File S1).

## 4. Malunion Manifestations

A femoral malunion may manifest as pain in the low back, hip, knee, ankle, or foot. This may significantly impair the patient’s mobility and even manifest as a limp. There may be limitations in knee and hip range of motion as significant as stiffness, resulting in uneven load transfer and restricted activity [5,19,23]. In some cases, the deformity can be severe enough to cause a troublesome cosmetic appearance. People have an inherent tolerance to deformity and may still be able to ambulate with shortening, malalignment, and malrotation. Articular malunion, however, is poorly tolerated and results in pain, stiffness, and limp with progression to post-traumatic arthritis with even small incongruencies.

## 5. Surgical Indications, Procedures, and Outcomes

The indications for surgical correction of femoral malunion include the development of clinical symptoms such as pain and limping, as well as the cosmetic presence of limb deformity. Deformity may be well tolerated, especially leg length discrepancy, and can be remedied with a shoe lift, thus rendering the problem asymptomatic for the patient. Ultimately, the decision for surgical intervention is made based upon a combination of history, physical examination, and radiographic evaluation. If the patient expresses dissatisfaction with their level of dysfunction or deformity, a surgical correction may be indicated [4,23,24,25,26,27].

When correcting a symptomatic malunion, one must have a plan to correct the deformity while ensuring that the biological healing environment facilitates union. The surgical approach and corrective procedure are generally custom-tailored to address the patient-specific malunion site and resulting deformity. Here we review the literature on femoral malunion correction strategies at individual anatomic regions of the femur in order to better illustrate and understand the inherent variability in management [4,5,23,24,26,27].

## 6. Femoral Head

Malunion of the femoral head is a relatively rare occurrence with only a few reports within the literature. Femoral head fractures (pipkin fractures) involve the articular surface of the hip joint, which makes restoration of a congruous joint surface a primary goal in most surgical interventions. Failure to restore this congruity can result in bony mechanical block and/or hip pain, femoroacetabular impingement, osteoarthritis, and osteonecrosis. Small bony fragments can be excised [28], while larger fragments can be osteotomized and re-fixed as open [29] or by arthroscope [30], with moderate success reported in two cases. Total hip arthroplasty is a good alternative in older patients.

## 7. Femoral Neck

Acute femoral neck fractures are generally treated with surgical intervention consisting of open or closed reduction and internal fixation in physiologically young patients, with arthroplasty reserved for physiologically older patients. Near-anatomic reduction of these fractures with screw fixation is considered necessary to allow for adequate bone healing, largely owing to a lack of local periosteum providing support during fracture callus formation [31]. Acceptable reduction parameters for these fractures have been suggested as <10 mm displacement, and <20 degrees of angulation in any plane [32,33]. There is a paucity of literature addressing the surgical correction of femoral neck malunion. This may be related to a lack of clear-cut parameters for appropriately defining clinically relevant malunion or because good clinical outcomes are usually achieved if the fracture heals despite a malunion being present [34,35].

### Peritrochanteric/Intertrochanteric

Intertrochanteric femur fractures are very common acute injuries, yet few articles address treatment of malunion in these cases. The reasons for this may be threefold: (1) most surgeons are adept at taking care of these injuries, leading to few malunions; (2) malunions occur relatively frequently but patients tolerate them well; or (3) malunions occur relatively frequently but symptoms are under-reported or ignored in these older individuals. Intertrochanteric fracture malunion is typically caused by either a neglected fracture or a failed surgical intervention. Evidence of infection in the presence of failed hardware necessitates hardware removal, necrotic tissue debridement, and culture-sensitive antibiotics [36]. In younger patients, the proximal fragment is often in better condition than in older patients, allowing for revision with internal fixation for the younger population and hip arthroplasty for the older population [37]. The femoral deformity caused by an intertrochanteric fracture malunion typically includes varus with a shortened leg length, recurvatum in the sagittal plane, and some degree of malrotation [36,37,38,39].

**Figure 3:**
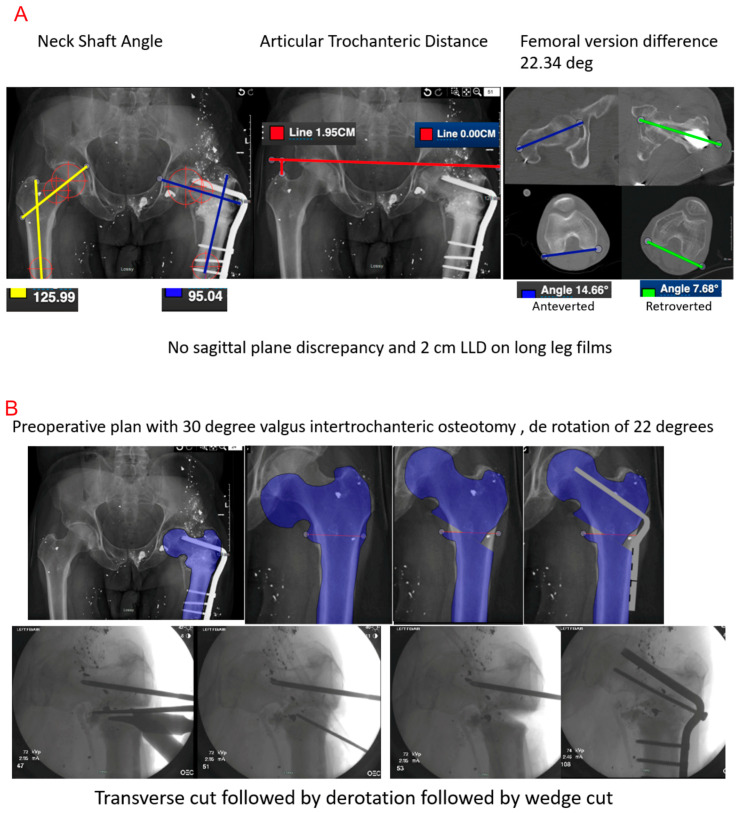

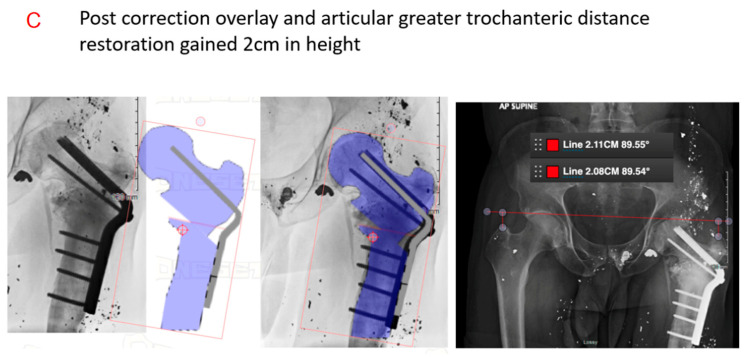
Stepwise correction of intertrochanteric femoral malunion using valgus intertrochanteric osteotomy (VITO). (**A**) Preoperative radiographs demonstrating varus deformity with a decreased femoral neck–shaft angle (125.99°), altered articular trochanteric distance, and femoral version difference of 22.34°. (**B**) Preoperative plan showing a 30° valgus intertrochanteric osteotomy with 22° derotation, performed via a transverse cut followed by wedge removal. (**C**) Post-correction images illustrating restoration of femoral version, neck–shaft alignment, and trochanteric height, resulting in anatomic reconstruction and 2 cm limb length restoration.

Valgus intertrochanteric osteotomy (VITO) is the preferred surgical procedure for simultaneous correction of varus deformity and leg length discrepancy with the goal of achieving an anatomic femoral neck–shaft angle, leg length, and femoral version. Surgery may involve removal of a wedge of bone to correct for leg length discrepancy, combined with deformity characteristics (Figure 3) [20,37,38,39]. The wedge width is determined by the quantitative value of limb shortening [37]. Other parameters that are important to consider are restoration of femoral version and articular trochanteric distance. Fixation can be achieved by sliding hip screw (SHS), cephalo-medullary nailing (CMN) or angled blade plates (ABPs) [20,37,38,39]. Postoperative care was not uniform between authors, and various papers described immediate mobilization with tolerated weight-bearing while others prescribed delayed mobilization and rehabilitation. However, each achieved similar outcomes with differing patient populations and biomechanics, suggesting postoperative care should be patient-tailored to achieve optimal results. The outcomes seem to be uniformly positive experiences, with patients achieving union and average Harris Hip Scores of 92 (76–98) [37]. Across the literature, all outcomes where the reported neck–shaft angle was corrected to at least 130 degrees with an increased range of motion tended to coincide with better outcomes [20,37,38,39] (Table 1) (Figure 3A–C).

## 8. Femur Shaft Malunion

Modern surgical fixation of acute femoral diaphysis fractures provides reliably successful fracture union in 99% of cases [1,2,3,4,5]. Unfortunately, this has been associated with a >10% rate of healing in a misaligned position in the coronal or sagittal plane, with rotational deformity reported in up to 30% of cases [40]. Comminution of the fracture makes reestablishing the length and rotation difficult, ultimately leading to unacceptable deformity in over 20% of cases [41,42]. A variety of surgical methods have been attempted for correction of femoral shaft malunion [18,24,43,44,45,46,47,48,49]. The generally accepted radiographic definition for these malunions includes >10° angular deformity, >10° rotational deformity, and/or >1.5–2 cm shortening (lengthening is unusual) [50,51,52,53,54,55,56,57,58,59,60,61,62]. Unlike cases of nonunion, patients with femoral malunion can usually ambulate and occasionally continue working without walking aids [18]. This may be a leading reason for the overall paucity of articles and cases published on this topic.

Delineating the parameters of the true femoral deformity is critical when constructing a plan for correction. Standard anteroposterior and lateral radiographs are useful in assessing the plane of the deformity while oblique views can supplement measurements to establish the plane of maximal deformity [54,55]. Acquiring images of the contralateral limb is useful for comparison, as well as for reverse planning. Utilization of CT scans to superimpose the hip, knee, and ankle joints for comparison has proven advantageous in the visualization of rotational deformity, as described by Paley and Mast/Teitge [54,55].

A single-plane (alignment) deformity in these instances can be acutely corrected using the traditional methods of opening- or closing-wedge osteotomy in the correct plane at the malunion site, followed by plate fixation with or without bone grafting [18,24,43,48,49,50,51,52,53,54,55,56,57,58,59,60,61,62]. A single-plane axial malrotation can be reliably corrected by a transverse osteotomy supplemented by nail fixation; however, plate fixation has also been implemented with acceptable results [55,62].

Biplanar (alignment, rotation) deformities can be corrected with an opening-wedge transverse osteotomy at the malunion site followed by axial correction through derotation of the limb. This can alternatively be accomplished with a closing-wedge osteotomy as well, which theoretically facilitates better healing while sacrificing limb length gains. In these cases, the transverse osteotomy is completed initially, with rotational correction performed prior to the bony wedging in the correct rotational axis. Fixation can be accomplished with a plate, a nail, or an Ilizarov/hexapod-type external fixator [18,24,43,48,63]. A mathematical osteotomy for a bi- or triplanar deformity can be a good option, but requires extensive preoperative planning in order to design the osteotomy prior to plating [45].

**Figure 4:**
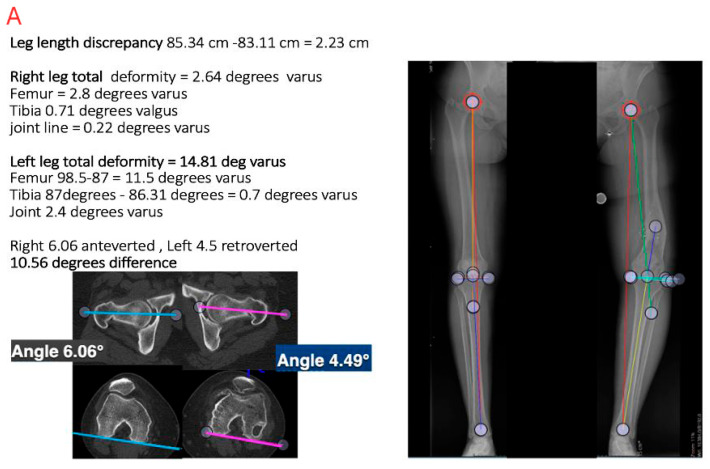

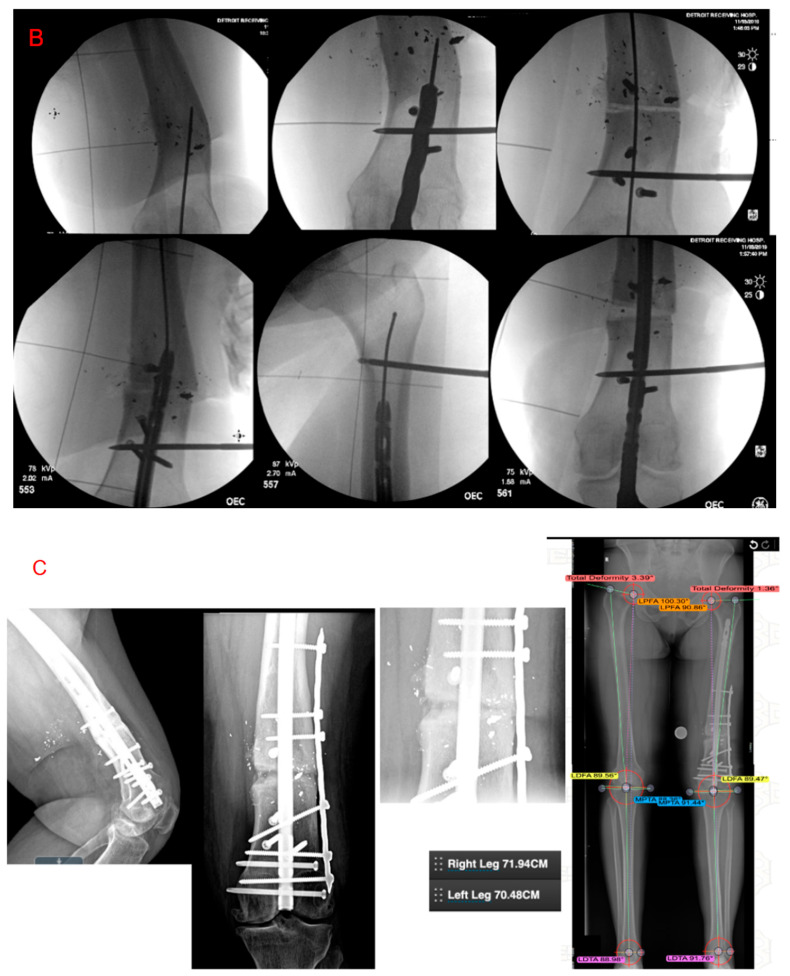
Acute correction of triplanar femoral shaft malunion using oblique osteotomy and intramedullary fixation. (**A**) Preoperative imaging demonstrating limb length discrepancy, varus alignment, and rotational malalignment. (**B**) Intraoperative fluoroscopic images showing oblique osteotomy and acute correction with intramedullary nail fixation. (**C**) Postoperative radiographs confirming restoration of mechanical alignment and limb length. Together, these images illustrate correction of length, angular, and rotational deformities achieved through a single-stage oblique osteotomy.

Triplanar deformity (length, alignment, rotation) can be acutely corrected via several methods. Limb length (up to 3 cm) may be gained through correction of the angular deformity [18,24,43,48,49,50,51,52,53,54,55] (Figure 4). Oblique osteotomies can provide an opportunity for increasing limb length, wherein the osteotomy is concurrently derotated while maintaining bony contact, or after introducing cancellous bone graft into the created gap following fixation [18,24,43]. The extent of acute lengthening is dictated by the surrounding soft tissues, with an upper limit of roughly 4 cm. Lengthening of this magnitude can be accomplished with an oblique or Z osteotomy [63] and nail fixation. Shortening osteotomy for LLD has rarely been described outside of a singular report by Winquist, who used an intramedullary saw to complete the osteotomy and address discrepancies in both length and alignment [62].

More recently, malunion correction with a novel clamshell osteotomy and intramedullary nailing has gained popularity. The osteotomy technique for this correction involves two transverse diaphyseal cuts made around the malunion site, with an additional cut along the diaphyseal long axis to connect the two. Postoperative follow-up has showed successful correction of limb length to within 2 cm and angulation to within 4°, with complete correction of rotational and translational deformity [49,50]. The correction is acute, can often accommodate acute lengthening up to 4 cm, and planning is minimized by simply fixing the bone in alignment using a nail.

Lengthening can also be achieved on a more gradual timeline. This involves treatment with an external fixator following Ilizarov guidelines; performing a corticotomy, waiting 7 days, and then performing distraction osteogenesis. For limb lengthening, osteotomy at the proximal or distal metaphyseal–diaphyseal junction is preferable. In the absence of angulation or translation, the distal metaphysis of the femur is the ideal site for length correction. The ideal location for corrective osteotomy tends to be that which is closest to the site of malunion. Translational deformity correction can be achieved by performing the osteotomy directly through the translation point. In the presence of multiple deformities, osteotomy is performed through the original line of malunion. This has the potential advantage of maintaining cortical bone overall during healing and the potential disadvantage of delaying healing since the osteotomy is being performed at a previous site of trauma [54]. Modern hexapod fixators have allowed for very precise correction when supplemented by correction planning software. This tool allows the surgeon to dial in the original correction or any residual correction to a perfect endpoint. Correction of rotational deformity is less dependent on a specific location and acceptable results can be reliably achieved at any anatomical segment. The drawbacks of this gradual correction technique include increased time requirement, potential pin site discomfort/complications, and presence of significant surrounding soft tissue (vs. the tibia). Despite the availability of multiple treatment options, the most important aspect during evaluation and treatment of these malunions is still properly defining the deformity while adhering to the principles of surgical corticotomy and distraction osteogenesis [64].

Finally, magnetic lengthening nails are a revolutionary device in deformity correction [65]. Similar to an Ilizarov apparatus, these allow acute correction of two-plane deformity while compensating for a shortened limb by using distraction osteogenesis with the implanted nail. Once the desired correction is achieved, the magnetic lengthening feature is easily halted. Additionally, any component of translation is automatically corrected by nature of the nail’s medullary fit [64]. This eliminates the presence of pin site complications which are common with external fixators.

Outcomes are good if thorough planning and a good correction can be achieved. Healing rates for distraction osteogenesis are seemingly identical when comparing techniques of lengthening nail vs. Ilizarov-type external fixation. Both methods allow for 0.75–1 mm of growth per day during the distraction phase, followed by allowance of roughly 1 month/cm growth for appropriate healing. So, a 3 cm lengthening would take 30 to 40 days to lengthen and then 3 more months for consolidation [54].

Closer analysis of cases of symptomatic femoral shaft malunion included in this literature review, wherein the deformity is clearly defined, describe angular or rotational deformities between 10 and 40 degrees. There were no corrections for cases with less than 10 degrees of angular or rotational deformity in our search.

While many of the reported complications associated with correction of femoral shaft malunion are mild-to-moderate in severity, one of the more extreme complications to be aware of is complete occlusion of the superficial femoral artery. This is a rare complication which reportedly occurred in an angular and rotatory correction [53]. Occlusion can lead to local soft tissue necrosis and overall poor surgical outcomes, thus general awareness and assessment of potential warning signs of this complication is beneficial in any correction, whether acute or gradual (Table 2).

## 9. Distal Femur

Distal femur fractures are at high risk of experiencing malunion, mainly due to the presence of deforming forces from surrounding muscular attachments to the distal fragment. These deforming forces typically manifest as shortening, apex–posterior angulation [52], and varus malalignment of the distal fragment. These fractures are also frequently comminuted, which significantly increases the difficulty in assessing normal anatomical alignment [23,26,27,52,59,60]. Restoration of near-anatomic alignment is especially important in these cases since up to 5 degrees of varus collapse can be seen following fixation with isolated, lateral-based implants. Diagnosis is made with a complete radiological workup with standing long leg and lateral X-rays to determine the mLDFA or aLDFA, the aPDFA, and LLD, with CT scans for axial alignment. One must take into consideration all three axes to correct, and this can be achieved with an opening [27,58] or closing wedge [23,26], either uniplanar or biplanar [26]; a dome [60]; or a clamshell [51]. Fixation is typically accomplished with either a single or a double plate [23,26,27,59], a nail alone, or a nail–plate combination [59,60].

Fractures with intraarticular involvement are typically fixed with the goal of complete anatomic reduction. Ozan et al. [21] and Sasidharan et al. [61] both reported on correction of a malunited medial femoral condyle Hoffa fracture. Malunion was identified radiographically by an incongruous femoral knee joint surface, and correlated clinically with a painful antalgic gait, and limited knee motion, a flexion contracture, and varus deformity. An open intraarticular corrective osteotomy was utilized for deformity correction held in place with screw fixation. The authors confirmed congruity of the articular surface postoperatively with a CT scan. Clinical improvements were noted as increased knee motion from 5° to 110° and painless weight-bearing. While these individual cases show that corrective procedures for intraarticular distal femur fracture malunions can result in good outcomes, larger studies are needed in order to make any definitive conclusions (Table 3).

### Complications

The literature reviewed in the current study encompassed several reviews and 33 case series, totaling 241 individual cases [18,20,21,23,24,26,27,28,29,30,35,36,37,38,39,40,43,44,48,49,51,52,53,54,55,56,58,59,60,61,62,63,66]. Many of the case series did not evaluate deformity as thoroughly as described in this paper, which can likely be attributed to the fact that the evaluation of femoral malunion is also evolving.

Closer scrutiny of these results revealed that there were 30 reported complications attributed to femoral malunion alone. The most reported complication was hardware failure/loss of correction (10). These were solved by grafting and adding more robust hardware universally with a successful outcome. Infection was reported in eight cases, which was treated with repeated surgery and antibiotics, again with successful outcomes. Pin tract infections were reported by Paley with external fixation but did not result in long-term sequelae [54]. One case of femoral artery occlusion due to an acute angular and rotational deformity is important to note. Treatment of these complications leads to decent outcomes. A few cases of mortality were reported but were unrelated to the surgery.

While clinical outcomes are often reported simply as “excellent” or “good” in early studies on femoral malunion correction, a majority of the literature since 2010 has included patient-reported functional outcome scores. The use of videography to document gait disturbances has also become more prevalent as smartphones become universally available. Much of the literature describing techniques for correction of femoral malunion reports excellent outcomes with high patient satisfaction with the procedure. As we continue to improve upon fracture treatment and amass more substantial amounts of patient-reported outcome measures (in contrast to traditional methods which primarily relied on radiographic monitoring of bony healing), the expectations for clinical outcomes following surgical correction are elevated for surgeons and patients alike. For example: residual LLD of up to ~4cm had historically been acceptable following surgical correction of femur fractures. Expectations have adapted accordingly with advancements in imaging, implants and technique, wherein patients in the modern age may complain of LLD ≥ 1.5 cm following surgery.

## 10. Limitations

The primary limitation of this review is the marked heterogeneity and scarcity of available data across studies. The included reports vary widely in anatomical location, surgical technique, fixation method, and outcome reporting, with most consisting of small case series or individual reports. As a result, no meaningful comparative analysis between surgical techniques could be performed. Attempting to draw direct comparisons from such disparate data would risk oversimplification and misrepresentation of clinical outcomes. Future studies employing standardized methodologies and outcome measures are necessary to enable reliable cross-technique comparisons. Furthermore, the predominance of Level IV and V evidence (case series and case reports) limits the overall reliability and generalizability of the findings. Prospective multicenter studies with uniform radiographic and functional assessment protocols are needed to validate these conclusions.

## 11. Conclusions

Correction of femoral malunion has been an evolving topic within the literature since its introduction decades ago. The definition of femoral malunion has evolved over time. Unlike nonunion, malunion reports are sparse in the literature. Correction is reserved for symptomatic individuals. Defining deformity by exam and radiological workup is important. Each individual femoral site is unique in its anatomic and biomechanical properties, such that they warrant individual considerations for reduction and fixation. Surgical correction of a malunion requires extensive preoperative planning to achieve the best possible results. The severity of the patient’s condition affects surgical outcome and return to preinjury condition, which has not been well reported within the current literature. Although the literature reports that the incidence of femoral malunion can be as high as 30%, most current studies on the topic include less than twenty subjects, which displays the need for continued reporting on malunion of the femur. The current corrective methods have shown promising outcomes, with complications focusing around low rates of nonunion that are solved by repeat surgery with grafting and increasing the stability of the fixation; infections treated by debridement antibiotics; and many reports lacking long-term outcome data and outcome measures.

For practicing orthopedic surgeons, accurate preoperative characterization of deformity using comprehensive radiographic evaluation (including full-length standing radiographs and 3D CT reconstruction) is essential. Preoperative templating and mechanical axis planning should guide correction strategy, with attention to restoring both anatomical and functional alignment. When planning corrective osteotomies, surgeons should also consider the patient’s functional goals, comorbidities, and soft tissue condition to minimize complications and optimize outcomes.

Looking forward, future research should focus on developing consensus deformity thresholds, standardized classification systems, and validated outcome measures to enable comparative synthesis of surgical techniques. The integration of computer-assisted planning, patient-specific instrumentation, and magnetically controlled intramedullary devices holds significant promise for improving precision and reproducibility in femoral malunion correction.

## Figures and Tables

**Table 1:** Reported clinical outcomes following surgical correction of proximal femoral malunion.

Study	Patients (n)	Surgical Treatment	Outcomes
Yoon et al. 2003 [28]	3	Partial femoral head ostectomy	Union eventually achieved in all cases, with return to activity and painless near-normal range of motion
Ross et al. 2012 [29]	1	Open corrective osteotomy, femoral head	Improved functional outcome score, with full return to activities and no residual motion deficits
Matsuda et al. 2014 [30]	1	Arthroscopic osteotomy of femoral head with bone grafting, screw fixation, and cephaloplasty	Return of preoperative motion, gait, and activity level, and improved functional outcome score
Verma et al. 2015 [35]	1	Non-operative treatment, displaced femoral neck	Successful union through callus formation. Full restoration of hip motion, complete absence of pain, and no restrictions during activity
Butala et al. 2016 [39]	5	Lateral closed wedge valgus osteotomy	All patients achieved radiographic union; femoral neck–shaft angle was corrected to 130 degrees or greater for every patient; average Oxford Hip Score 39
Subash 2011 [38]	15	Valgus osteotomy with DHS fixation	All patients healed without complications; improved mean Harris Hip Score from 72.33 (61–80) to 91 (80–97); and nearly anatomical abduction, adduction, flexion, internal rotation, and external rotation
Bhowmick et al. 2020 [20]	12	Valgus osteotomy	All patients healed without complications with either absent or mild pain; average Parker Mobility Scale score 8.25 (9–5)
Bartonícek et al. 2003 [37]	11	Valgus intertrochanteric osteotomy	All patients achieved radiographic union with one patient requiring revision surgery and two delayed union; average Harris Hip Score increased to 90.2 (76–98) from 75 (65–82)

Summary of published studies detailing patient characteristics, surgical techniques, and postoperative outcomes for femoral head, neck, and intertrochanteric malunions. Valgus intertrochanteric osteotomy (VITO) and related procedures consistently demonstrated high union rates and improved functional scores, particularly when the femoral neck–shaft angle was corrected to ≥130°. Functional outcome measures: Harris Hip Score (HHS, 0–100; higher = better function), Oxford Hip Score (OHS, 0–48; higher = better function), and Parker Mobility Scale (PMS, 0–9; higher = better mobility).

**Table 2:** Reported studies on surgical correction of femoral shaft malunion and associated complications.

Study	Patients (n)	Deformity	Surgical Treatment	Outcomes	Complications
Knight et al. 1980 [53]	1	35 degrees anterior angulation; 25 degrees external rotation	Osteotomy with Kuntscher nail	Anatomic correction	Occlusion sup femoral artery
I. Kempf et al. 1986 [63]	15	12 LLD plus axial 3	Acute lengthening up to 4cm with 13 Z osteotomies, 1 oblique, 1 transverse/12 lengthening 3 length rotation	Healed some loss of length	3 deep infections and 2 losses of correction
Winquist 1986 [62]	12	Angular not described	Angular deformities osteotomy plus bone graft if an open wedge occurs	10 /12 anatomic	2 incomplete corrections
Paley et al. 1990 [54]	6	Short >1.5 cm angulation >5 deg Rotation >15 deg translation >1 cm	Percutaneous Osteotomy and Illizarov apparatus	All corrected	Minor pin tract infections
Mast et al. 1990 [55]	12	0–3.5 cm short, 15 val to 20 var 5 pro to 35 recurvatum, 0–30 deg ir	Osteotomy closing or open wedge with plate	All reduced within acceptable parameters	N/a
Farquharson-Roberts 1995 [43]	1	Rotational deformity >10°, LLD >1.5 cm, shortening >3.5 cm; external rotation deformity ~30°	Oblique rotational osteotomy/IM Nail	Complete correction of rotational and angular deformity within 1 cm	N/a
Wu et al. 2001 [18]	21	Angulation 24 + 6 deg 18 pts/malrotation 25–30 deg in 3 pts shortening of >2 cm 10 pts	Oblique rotational osteotomy/IM Nail	Restored knee ROM and deformity, healed to normal parameters (angulation < 10°; malrotation < 10°; shortening < 2cm)	N/a
Chiodo et al. 2003 [66]	6	Average varus 21.7° (range, 12°–32°); average antecurvatum 22.8° (range, 10°–30°); average leg length discrepancy was 1.8 cm (range, 0.5–3 cm	Osteotomy with ORIF Plate	Average varus deformity improved from 21.6 degrees to 4.2 degrees. The average deformity improved from 22.5 degrees to 7.0 degrees antecurvatum	N/a
Lammens et al. 2008 [48]	1	2 cm shortening; unspecified amount of antecurvatum	Ilizarov percutaneous osteotomy/removal of prior nail	Anatomic correction	N/a
Russell et al. 2009 [51]	4	Unspecified/non-quantified deformity	Clamshell osteotomy with intramedullary nail	Complete correction of limb length inequalities to within 2 cm (0–5 cm)	N/a
Tall et al. 2012 [49]	16	Mean LLD of 3 cm (2–6 cm); mean knee flexion limitation of 90°	Oblique rotational osteotomy IM Nail	Healed to normal parameters (angulation < 10°; malrotation < 10°; shortening < 2cm)	N/a
Middleton et al. 2018 [24]	7	Average shortening of 2.7 cm. 5/7 had average rotational deformity of 33°. 2/7 with tri-planar deformity (vs. biplanar in 5/7	Oblique rotational osteotomy plate graft	Healed to normal parameters (angulation < 10°; malrotation < 10°; shortening < 2 cm)	N/a

Summary of published case series and reports describing deformity patterns, surgical techniques, outcomes, and complications following correction of femoral shaft malunion. Across studies, osteotomy techniques—including oblique, Z-, and clamshell osteotomies with plate or intramedullary fixation—consistently restored alignment, rotation, and length to near-anatomic parameters. Complications were infrequent, with superficial femoral artery occlusion and infection being the most notable events. Functional outcome measures: Improvements in range of motion (ROM) and reduction in limb length discrepancy (LLD) indicate better postoperative function and alignment.

**Table 3:** Reported outcomes of surgical correction for distal femoral malunion.

Study	Patients (n)	Deformity	Surgical Treatment	Outcomes
Gugenheim et al. 2003 [59]	2	Mechanical axis deviation of >15 mm; mLDFA <85° or >90° 90 mm lateral to 120 mm medial	Temporary external fixation and subsequent percutaneous dome osteotomy retrograde nail	Correction of mLDFA and mechanical axis Average mLDFA ~89° MAD <15
Chou et al. 2008 [60]	1	15° of coronal plane varus and 8.7° within the sagittal plane, mechanical and anatomical axes in 8° and 3° of varus	Navigation-assisted TKA	Resolution of pain and LLD; ROM improved Radiographically normal knee alignment
Wu et al. 2014 [58]	24	aLDFA bw 2 and 14 deg varus	Opening-wedge osteotomy Blade plate or nail multi-planar deformity	Ideal knee alignment and knee function 4.2 mo healing time, 4 nonunions 2 infected, repeat surgery healed them correction to within 3 deg of aLDFA 79–82 deg
van der Woude et al. 2016 [26]	5	Non-zero mFTA; MPTA and mLDFA <85° or >90°; knee JLCA >3° medially	Lateral closing wedge valgus osteotomy single plane deformity uni- or biplanar osteotomy	VAS scores improved with maintained range of motion biplanar healed faster average correction of preoperative mFTA from 10.0° (±2.6°) of varus to 3.1° (±2.6°) varus postoperatively, and of mLDFA from 95.9 (±2.7) to 89.3 (±2.9), with no significant changes in MPTA or JLCA
Sasidharan et al. 2016 [61]	1	Incongruous femoral knee joint surface	Open intraarticular corrective osteotomy	Improved ROM Complete anatomic alignment 20–80 deg to 5 to 110 deg
Ozan et al. [21] 2018 [21]	1	Incongruous femoral knee joint surface	Open intraarticular corrective osteotomy	Improvements in function and pain, with return to normal activities Complete anatomic alignment 25 deg flexion contracture improved ROM and function
He et al. 2019 [27]	15	Non-zero mFTA; mLDFA <87° or >90°, aPDFA of <79° or >87°; LLD >2–3 cm	Medial open-wedge osteotomy with double plate fixation	LLD improved to 0.8cm; VAS scores increased mFTA from 17.5° preoperatively to 2.3° postoperatively; mLDFA from 102.3° to 85.2°; aPDFA from 77.1° to 82.7°; and LLD from 3.38 cm to 0.8 cm
Rollo et al. 2019 [23]	22	mLDFA <85° or >90°in the coronal plane, PDFA <79° or >87° in the sagittal plane. Any rotational or intraarticular deformity. No specific radiographic parameters noted.	Lateral closed-wedge blade plate augmented with an auto- or allograft strut	Improved functional outcome scores had poor outcomes. Bony union with significant improvements in limb length discrepancy, quality of life, and knee functionality; no correctional parameters given

Summary of published studies describing deformity characteristics, surgical approaches, and postoperative outcomes for extraarticular and intraarticular distal femoral malunions. Various osteotomy techniques—including dome, wedge, and intraarticular corrective procedures—achieved significant improvements in mechanical alignment, joint congruity, pain, and function. Most series reported restoration of the mechanical lateral distal femoral angle (mLDFA) to near-normal values and substantial improvement in range of motion and limb length. Functional outcome measures: Visual Analog Scale (VAS, 0–10; lower = less pain), range of motion (ROM; greater = better joint mobility), and limb length discrepancy (LLD; smaller = better symmetry).

## Data Availability

No new data were created or analyzed in this study. Data sharing is not applicable.

## Supplementary Materials

The following supporting information can be downloaded at: https://www.mdpi.com/article/10.3390/medicina61112050/s1, File S1: Femoral malunion alignment parameters (PowerPoint). This supplementary presentation illustrates key radiographic parameters and measurement techniques used in the assessment of femoral malunion. Included diagrams demonstrate the relationship between the mechanical and anatomic axes, definitions of the mechanical lateral distal femoral angle (mLDFA), anatomic lateral distal femoral angle (aLDFA), and anatomic posterior distal femoral angle (aPDFA), as well as the evaluation of axial alignment on CT imaging. Each figure provides visual references for normal alignment ranges and deformity classifications described in the manuscript.

## Abbreviations

The following abbreviations are used in this manuscript:

LLD	Leg Length Discrepancy
MAD	Mechanical axis deviation
CT	Computed Tomography
AP	Anteroposterior
LDFA	Lateral distal femoral angle
PDFA	Posterior distal femoral angle
MLDFA	Mechanical lateral distal femoral angle
APDFA	Anatomic posterior distal femoral angle
MPTA	Medial proximal tibial angle
VITO	Valgus intertrochanteric osteotomy
SHS	Sliding hip screw
CMN	Cephalo-medullary nailing
ABP	Angled blade plates
OHS	Oxford hip score
PMS	Parker mobility scale
ROM	Range of motion
TKA	Total knee arthroplasty
VAS	Visual assessment scale
MFTA	Mechanical femoral tibial angle
JLCA	Joint line convergence angle
