# Supplementary material for: Femoral Malunion and Its Correction: A Review
Source: Medicina (Kaunas). 2025 Nov 17;61(11):2050. doi: 10.3390/medicina61112050 (PMC12654028; doi:10.3390/medicina61112050)
Supplement: Supplementary file 1 [file medicina-61-02050-s001.zip › medicina-3953525-supplementary.pptx]

## Slide 1
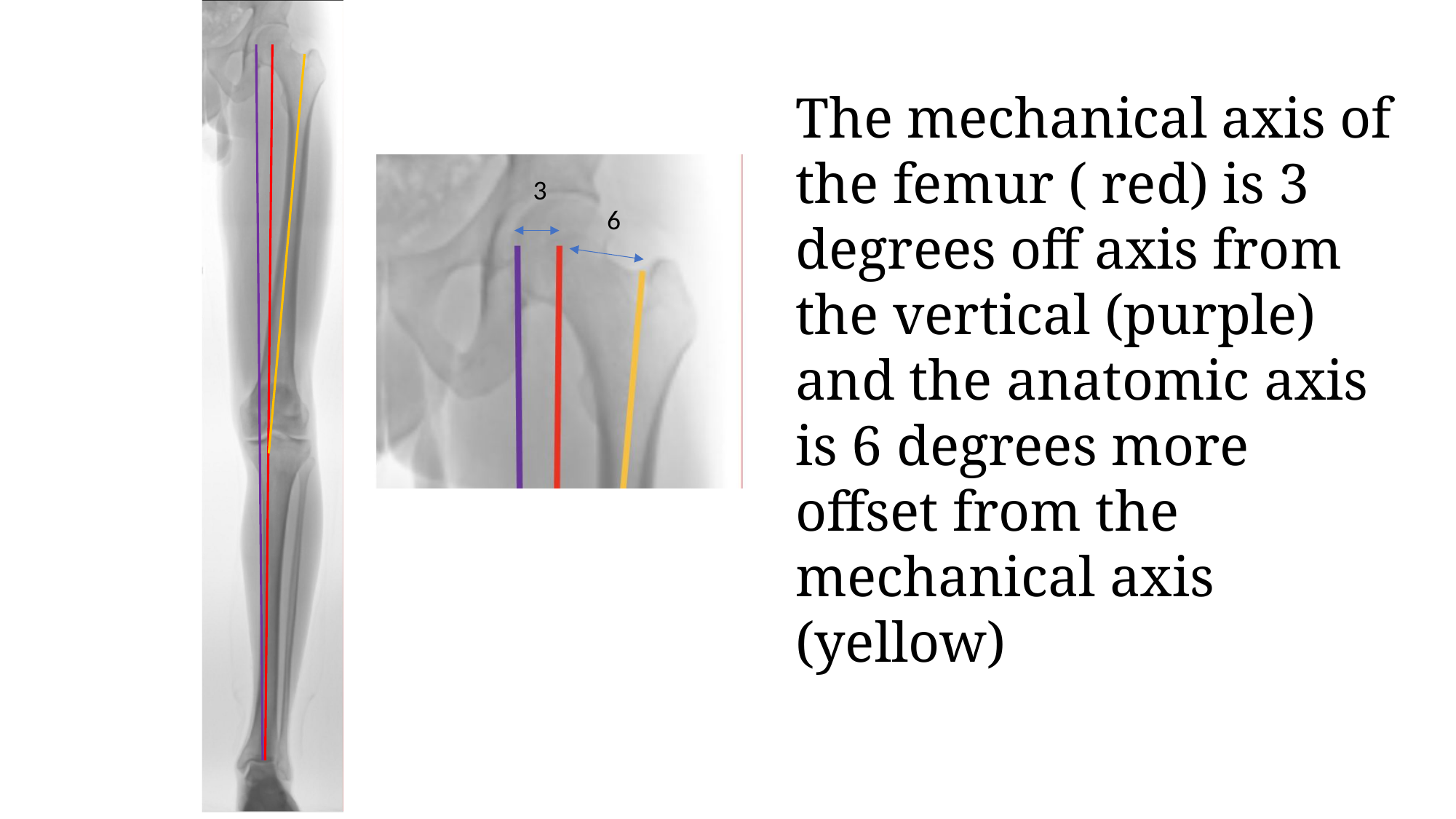

The mechanical axis of the femur ( red) is 3 degrees off axis from the vertical (purple) and the anatomic axis is 6 degrees more offset from the mechanical axis (yellow)
3
6

## Slide 2
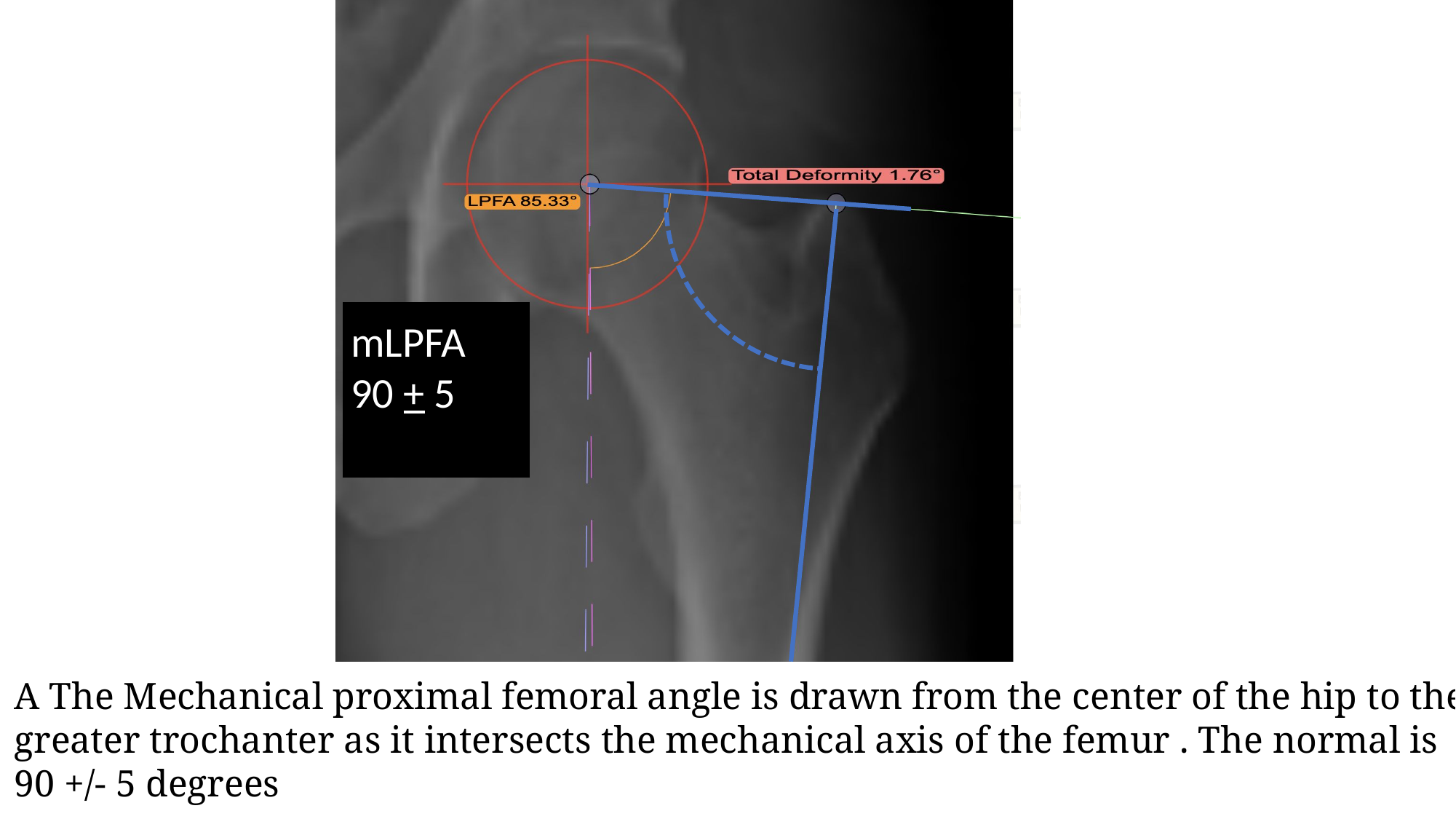

mLPFA
90 + 5
A The Mechanical proximal femoral angle is drawn from the center of the hip to the greater trochanter as it intersects the mechanical axis of the femur . The normal is 90 +/- 5 degrees

## Slide 3
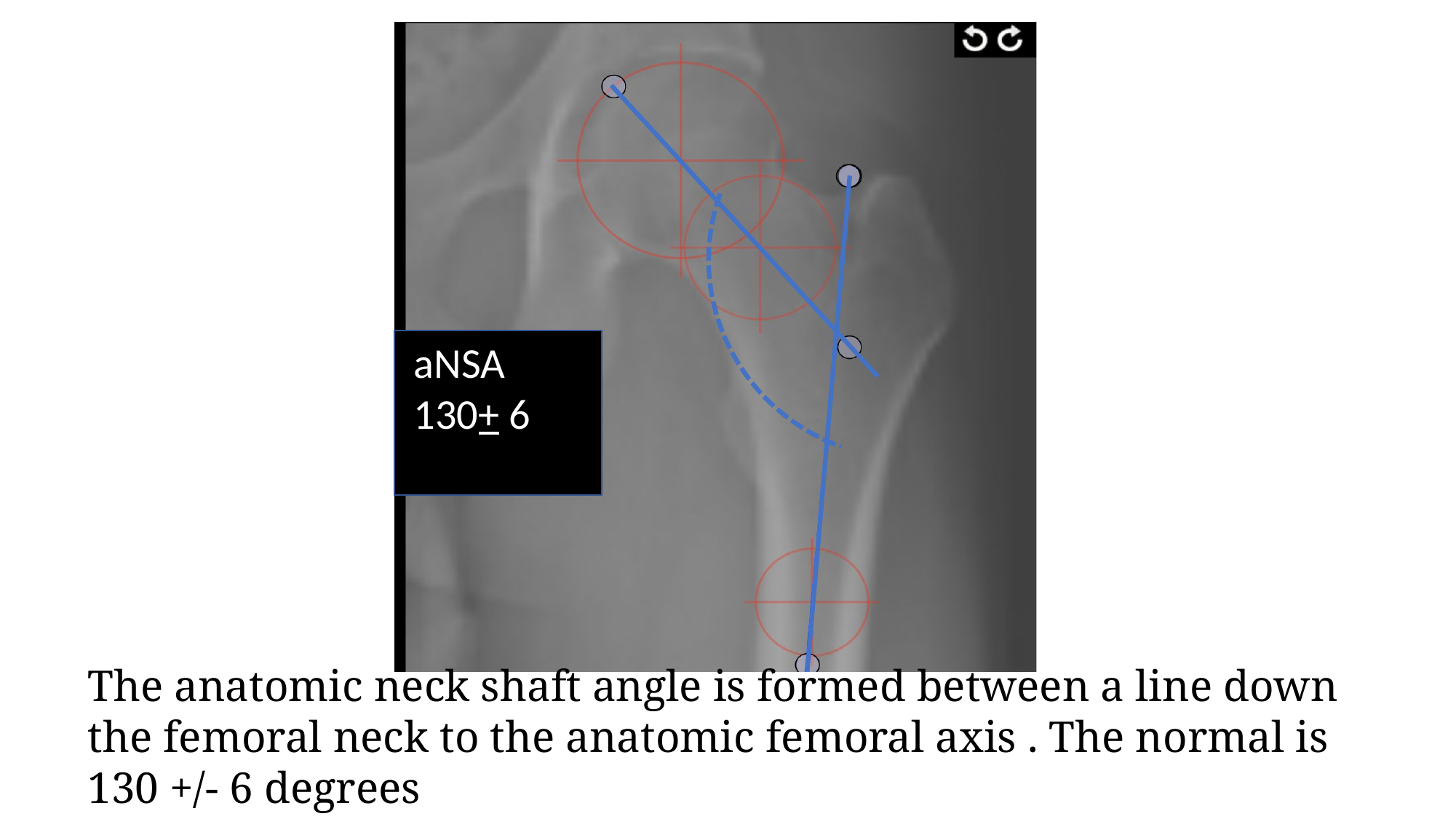

aNSA
130+ 6
The anatomic neck shaft angle is formed between a line down the femoral neck to the anatomic femoral axis . The normal is 130 +/- 6 degrees

## Slide 4
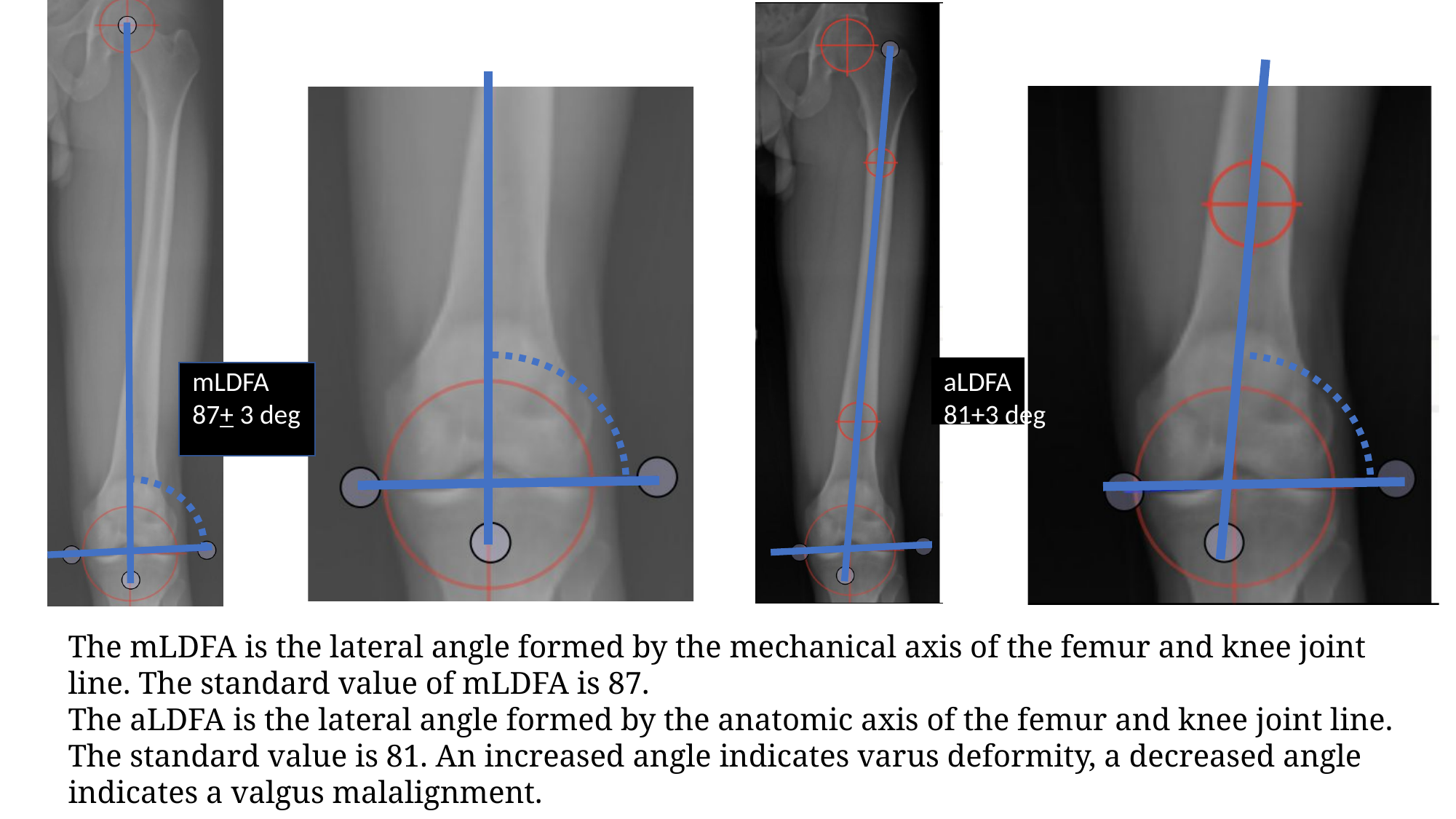

mLDFA
87+ 3 deg
aLDFA
81+3 deg
The mLDFA is the lateral angle formed by the mechanical axis of the femur and knee joint line. The standard value of mLDFA is 87.
The aLDFA is the lateral angle formed by the anatomic axis of the femur and knee joint line. The standard value is 81. An increased angle indicates varus deformity, a decreased angle indicates a valgus malalignment.

## Slide 5
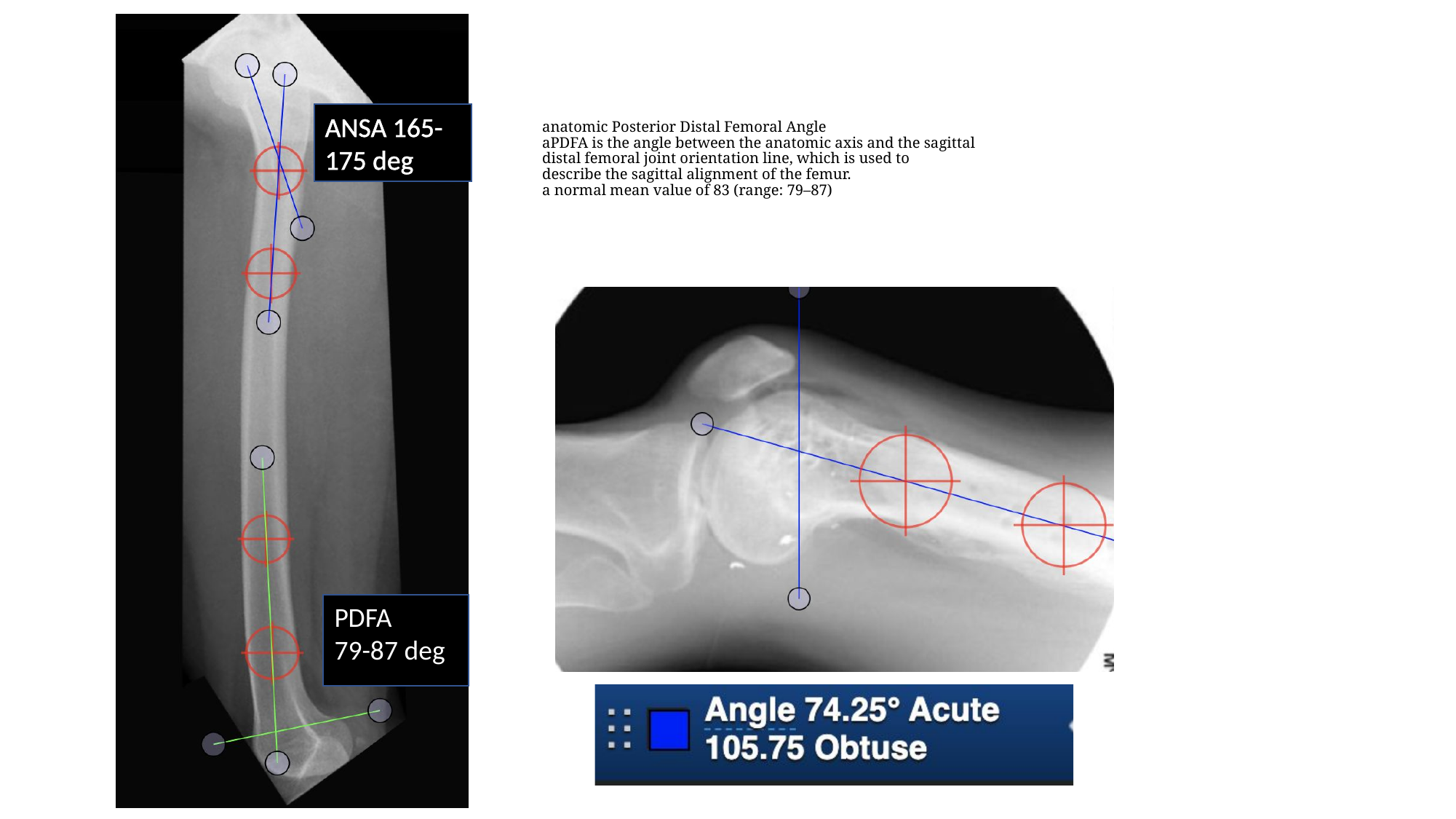

# anatomic Posterior Distal Femoral Angle aPDFA is the angle between the anatomic axis and the sagittal distal femoral joint orientation line, which is used to describe the sagittal alignment of the femur. a normal mean value of 83 (range: 79–87)
ANSA 165-175 deg
PDFA
79-87 deg

## Slide 6
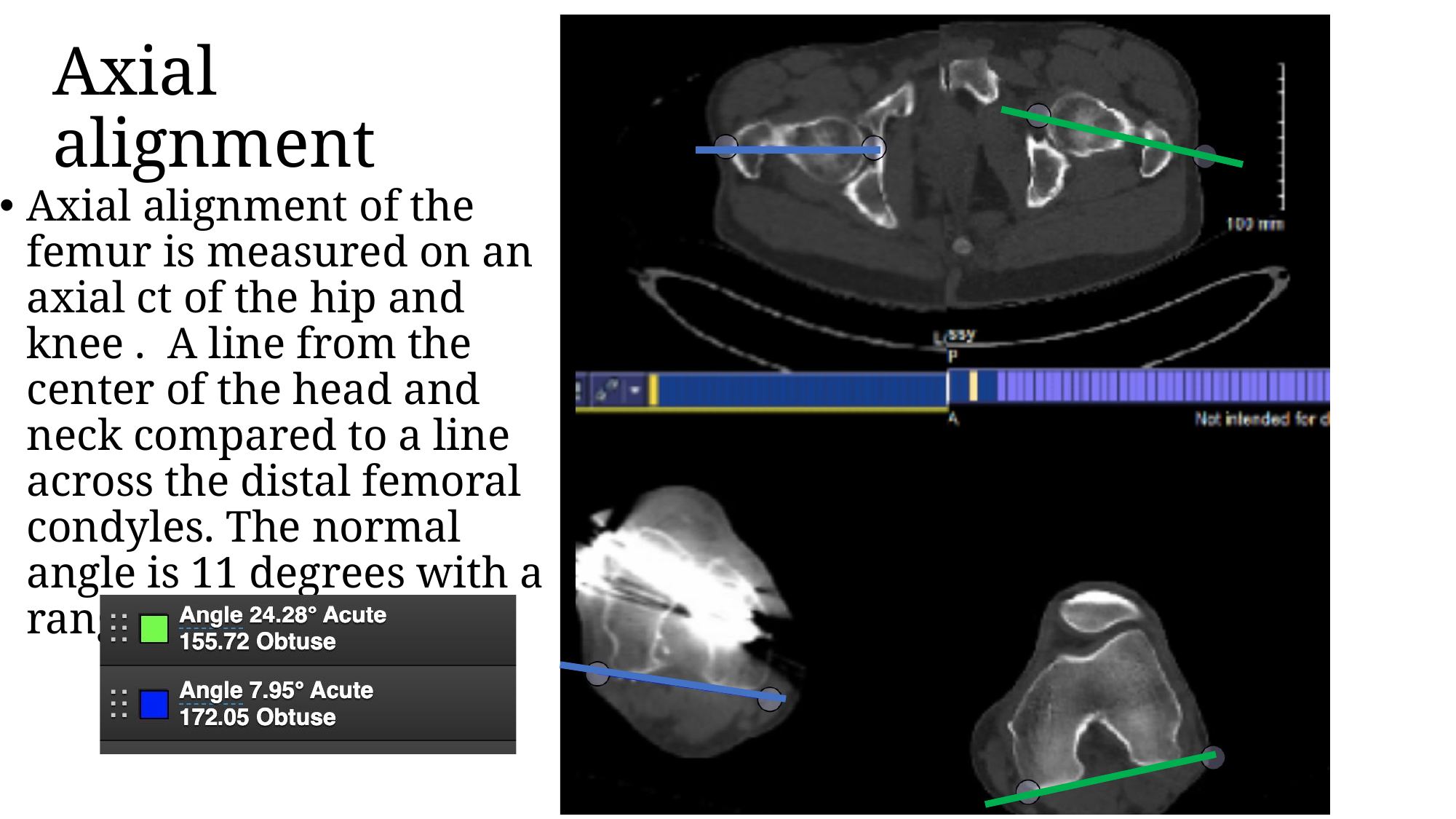

# Axial alignment
Axial alignment of the femur is measured on an axial ct of the hip and knee . A line from the center of the head and neck compared to a line across the distal femoral condyles. The normal angle is 11 degrees with a range of 5 to 15 deg
